# Assessment of calvarial structure motion by MRI

**DOI:** 10.1186/1750-4732-3-8

**Published:** 2009-09-04

**Authors:** William T Crow, Hollis H King, Rita M Patterson, Vincent Giuliano

**Affiliations:** 1 Florida Hospital East Orlando, 7975 Lake Underhill Road, Orlando, Florida 32822 USA; 2 The Osteopathic Research Center, University of North Texas Health Science Center, 3500 Camp Bowie Boulevard, Fort Worth Texas 76107 USA; 3 Department of Osteopathic Manipulative Medicine, University of North Texas Health Science Center, 3500 Camp Bowie Boulevard, Fort Worth, TX 76107 USA; 4 NOVA Southeastern University College of Osteopathic Medicine, VinCon MRI Center, 5732 Canton Cove, Winter Springs, FL 32708, USA

## Abstract

**Background:**

Practitioners of manual medicine/manual therapy (MM/MT) who utilize techniques thought to have some impact upon and move the solid structures of the human head have been criticized for lack of evidence of cranial bone motion. The present study utilized magnetic resonance imagery (MRI) technology to address the question of whether or not inherent (non-operator initiated) calvarial structure motion can be assessed.

**Methods:**

Subjects: Twenty healthcare professionals, (physicians, nurses, medical students, pharmacists) between the ages of 24 and 52 were recruited. Seven females (ages 25-47, mean age 36.7) and 13 males (ages 25-53, mean age 31.2) volunteered. Technology: MRI scans were acquired at 450 ms per slice, in a 1.5 Tesla Signa Excite HD closed MRI system. The same scan prescription was repeated serially every 45 seconds to obtain eight serial slices for each subject. Image analysis was accomplished using ImageJ software (ImageJ 1.33 u National Institutes of Health, USA). Data from all eight images for each of the 20 subjects were analyzed to determine the two images with the largest differences in the parameters measured.

**Results:**

Difference values for the measures of area, width, height, major axis, and feret were statistically different whereas the measures for perimeter and minor axis were not. However, only the difference values for area were both statistically different (p < 0.003) and exceeded the resolution threshold of 0.898 mm/pixel.

**Discussion:**

The statistically significant difference value for area is suggestive of inherent motion in calvarial structures, and adds to the body of evidence supportive of biomechanically measurable calvarial structure motion in general. That the total intracranial area appeared to expand and recede was consistent with theory and prior studies suggestive of calvarial structure motion due to intracranial fluid volume changes.

**Conclusion:**

The use of MRI technology was able to demonstrate calvarial structure motion at a level exceeding the resolution threshold, and provides a means for further research on phenomena related to the cranial concept. It may be just a matter of time until increased resolution of MRI technology and image analysis provide the ability to examine more detailed areas of specific cranial bone motion.

## Background

Practitioners of manual medicine/manual therapy (MM/MT) who utilize techniques thought to have some impact upon and move the solid structures of the human head have been criticized for the apparent lack of evidence for the capability of cranial structures to move, much less the mechanism of action for such possible motion. Research suggestive of cranial structure motion has been generated in the past decade by physiology and neuroscience researchers concerned with intracranial fluid dynamics [1-6]. National Aeronautics and Space Administration (NASA) supported research, along with research carried out by former Russian Cosmonaut program scientists [7-11] has increased the credibility and potential applicability of MM/MT as it relates to structures of the human head. Recent anatomic research suggested that calvarial sutures remain patent with the degree of patency dependent on the amount of muscular attachment on a particular calvarial bone and the activities of chewing and movement of the head upon vertebral column [12].

Studies on animal subjects have been conducted under conditions which allowed controlled calvarial bone motion production and observation. Adams et al [13] studied parietal bone mobility in adult cats. They used a multiplanar strain gauge to measure parietal bone motion in response to externally applied forces and to changes in intracranial pressure induced by artificial cerebrospinal fluid injected into the subarachnoid space. Measurable motion did occur, with the range being 17 to 70 microns. Lateral head compression induced sagittal suture closure and inward rotation of the parietal bones. Increased intracranial pressure induced a widening of sagittal suture and outward rotation of parietal bones, with the same effect produced by direct pressure on the sagittal suture.

On squirrel monkeys, Michael and Retzlaff [14] performed direct measurement of right parietal bone motion using a screw attachment and a displacement transducer. With the primate's head immobilized in a stereotaxic frame, bone displacement, mean arterial blood pressure, and heart and respiration rates were simultaneously measured. Spontaneous cranial motion and the effects of applying external forces and passive spinal motion were recorded. Results showed two patterns of spontaneous parietal bone motion. One pattern was synchronous with respiration rate. This was superimposed over a second, slower oscillatory pattern consisting of 5-7 cycles per minute that was not attributable to heart rate, respiration rate, or central venous pressure. Force applied to the skull in various locations generally produced motion between the parietal bones.

While not conclusive as to mechanism of action, the animal research showed that calvarial bone movement does occur and may be related to oscillations in physiologic functions such as heart rate and respiration rate. To date the only attempt to correlate calvarial bone motion with a physiologic impetus, in the context of the cranial motion theory of Sutherland known as the primary respiratory mechanism (PRM) [15,16], was by Moskalenko et al [11] who theorized a harmonic effect of vascular and neurological processes as the motive force of the PRM.

In order to establish a greater evidence base demonstrative of cranial/calvarial bone motion, which would then lead to research on the mechanism(s) of action, the utilization of imagery technology was selected. There is precedent for such a path of research. The utilization of x-ray imagery technology to assess cranial structure motion was done in a pilot study on humans [17], and suggested that MM/MT intervention may have the capability to alter cranial bone biomechanical relationships. While magnetic resonance imagery (MRI) technology was used in the Russian research [9,10], corroboration of their findings with a larger number of subjects is needed. Therefore, to further study the proposed and theoretically formulated model of inherent, intrinsic cranial structure motion [15,16], the use of MRI technology to assess cranial structure motion was carried out on healthy human subjects.

Utilizing MRI on healthy human subjects the hypothesis was that eight serial slices through exactly the same calvarial plane over a six minute period would show no deviation on any plane or vector. The null hypothesis was that there would be no difference in measurable dimensions for each subject.

## Methods

### Subjects

Twenty healthcare professionals, (physicians, nurses, medical students, pharmacists) between the ages of 24 and 52 were recruited. Seven females (ages 25-47, mean age 36.7) and 13 males (ages 25-53, mean age 31.2) volunteered. The age difference was not statistically significant (*p *= 0.15). Volunteers were excluded if they were pregnant, had a history of surgery of the cranium or face, had metal implants that would preclude use of the MRI, or the radiologist determined that it would be unsafe for the person to participate. This study was reviewed and approved by the Institutional Review Board of Florida East Hospital of Orlando, and the MRI studies were carried out in the Radiology Department of Florida East Hospital of Orlando, FL.

### MRI Capability and Utilization

In the present study Serial axial T1-weighted MRI scans were acquired at 450 ms per slice, in combination with a dedicated phased array head coil in a 1.5 Tesla Signa Excite HD closed MRI system (GE Medical Systems, Milwaukee, WI). A single axial T1-weighted scan slice was prescribed at approximate maximum mid-cranial diameter, at the level of the parietal bones as determined by multiplanar T1 gradient-echo localization. The same scan prescription was repeated serially every 45 seconds to obtain eight serial slices. The number of scans and interval between scans were arbitrarily determined as the rate of PRM motion varies between individuals [15,16]. The assumption was that a consistent time interval between scans would provide a stronger, more easily replicated research design, and was likely to provide images which would capture at or near maximum and minimum PRM flexion-extension excursion [15,16].

**Figure 1:**
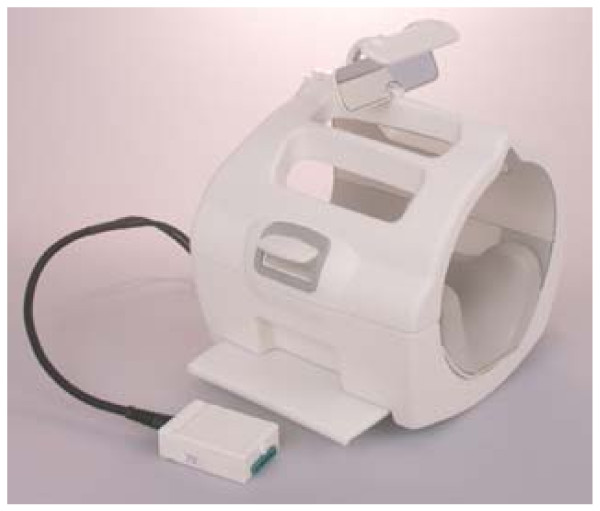
**GE Medical Systems 1.5 Split Head Coil Mount**.

**Figure 2:**
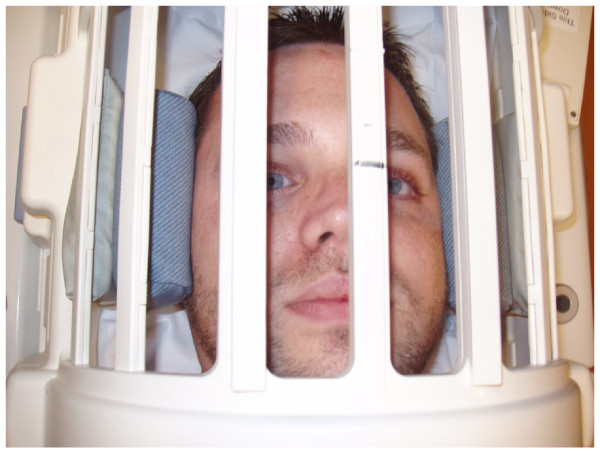
**Head mount with human subject in place**.

The MRI protocol, including equipment and technology, was reviewed by the Scientific Review Committee of Florida East Hospital of Orlando, FL which included two radiologists, one of whom specialized in neuroradiology. The radiologists specifically required the use of the GE Medical Systems 1.5 Split Head Coil head mount which met the current standard of practice for elimination of head movement (Figures 1 and 2). The use of this particular head mount raised the concern that the very tight fit on subject's head could reduce or eliminate any possible calvarial motion, however this precaution was required for the project to be approved.

**Figure 3:**
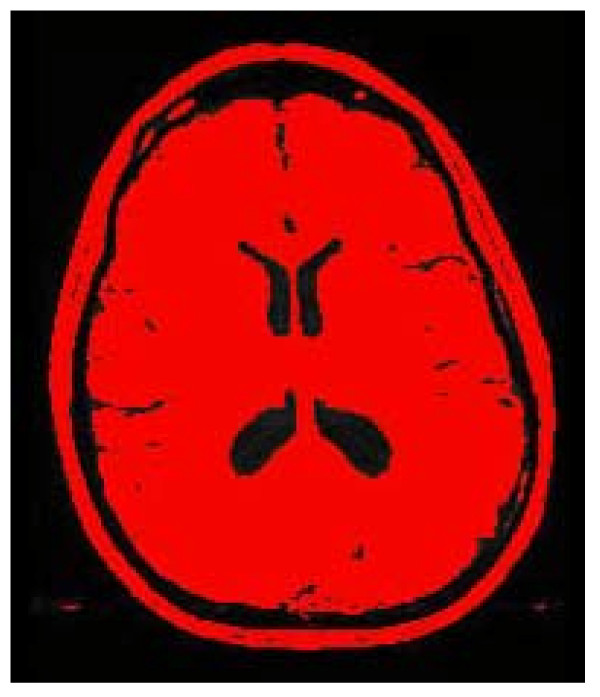
**Threshold image**.

**Figure 4:**
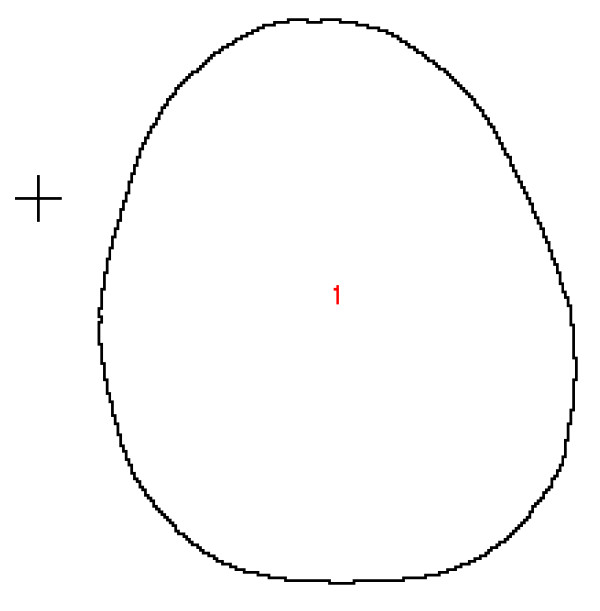
**Threshold outline**.

Two dimensional MRI scans, 23 centimeters high and 23 centimeters wide were obtained. Each 2D MRI image contains an array of 256 pixels wide by 256 pixels high. Thus, the image resolution in this case is 0.0898 cm/pixel (23 cm divided by 256 pixels and converted to mm is 0.898 mm/pixel). The images were saved in DICOM format and then converted in TIFFs for importation into ImageJ software (ImageJ 1.33 u National Institutes of Health, USA) for evaluation. The images were thresholded to interactively set lower and upper threshold values between 40 and 255. Figure 3 is representative of the image produced by this step of ImageJ analysis. Then the threshold image was analyzed using the analyze particles command in ImageJ. The minimum pixel size was set at 10 and maximum to 999999 in order to obtain the external contour of the image. Figure 4 is representative of the image produced by this step of ImageJ analysis. Area, perimeter, height and width of a bounding rectangle, major and minor axes of the best fit ellipse, and the feret diameter (longest distance between any two points along the boundary) were calculated using the analyze particles function in ImageJ. Data were imported into an Excel^® ^spreadsheet for analysis. Data from all eight images for each of the 20 subjects were analyzed to determine the two images with the largest differences in the parameters measured. The differences between these two images were recorded and means determined for all 20 subjects.

The study was completed in one day on the same machine. Each subject's head was positioned in the mount as shown (Figures 1 and 2). The head was cradled in the firm rubber device which fit tightly under the cranial base and slightly overlapped the occipito-parietal bone suture. With the subject's head in the head mount, the mount was secured to the table, and subjects were instructed to lie as still as possible for the six minute MRI procedure. Each subject was visually monitored for the entire six minute procedure and there appeared to be no body or head movement at any time by any subject. There were no instances of coughing or other reflexive behaviors which could have caused head movement.

## Results

Statistical analysis using a two tailed paired t-test with hypothesis of a difference of zero was performed. Table 1 displays the mean ± the standard deviation differences between the maximum and minimum values for all measures evaluated by ImageJ, confidence intervals and *p *values. Difference values for the measures of area, width, height, major axis, and feret were statistically significant, whereas the difference values for perimeter and minor axis were not statistically significant. While statistically different, the measures for width, height, major axis, and feret were below the resolution threshold of 0.898 mm/pixel and could not be used reliably to determine changes in cranial shape due to PRM. The difference values for area measure were both statistically different (p < 0.003) and were well above the resolution threshold of 0.898 mm/pixel.

**Table 1:** Mean difference between the maximum and minimum values for each of the measures evaluated by ImageJ for each subject (N = 20), using a two tailed paired t-test

**Measure**	**Measure Values (STD, %; CI)**	***P *value**
Area (mm)^2^	122.69 (75.84, 95%; 81.43 - 163.96)	0.003

Perimeter (mm)	4.00 (6.18, 95%; 0.63 - 7.36)	0.80

Width (mm)	0.49 (0.54, 95%; 0.19 - 0.79)	0.05

Height (mm)	0.63 (0.66, 95%; 0.27 - 0.98)	0.004

Major (mm)	0.67 (0.48, 95%; 0.40 - 0.93)	0.001

Minor (mm)	0.27 (0.19, 95%; 0.16 - 0.36)	0.08

Feret (mm)	0.70 (0.55, 95%; 0.40 - 0.99)	0.001

## Discussion

While just under resolution values of 0.898 mm/pixel, the statistically significant difference values for width, height, major axis, and feret may suggest changes in calvarial dimensions worth further examination under more precise and controlled technical conditions, such as with higher resolution MRI capability. Also further advances in the ImageJ technology may result in greater applicability in research designs comparable to the present study.

The statistically significant difference values for area, which were above the resolution threshold limits of the MRI technology available for use in the present study, suggests that calvarial structures may move independent of any external or internally applied forces in normal human subjects. Were it the case that calvarial structures were immobile as might be the case if cranial sutures were completely fused and the calvarial structures incapable of any deformation or boney compliance, then no changes in position, as results in the present study suggest, would be possible.

It was not assumed that the minimum and maximum differences for each dimension represented the full excursion of flexion and extension of the PRM. Nor was there any attempt to equate ImageJ dimensions with MM/MT cranial treatment terminology such as "height" with anterior-posterior calvarial axis, "width" with bi-parietal diameter, and "perimeter" with circumference. However, the calvarial slice image placement was purposely placed over the parietal, temporal and occipital bones, which clinically and in other studies [2-6,18,19] are reported to have the greatest amplitude of change.

The contention of the authors is that the statistically significant difference between the minimum and maximum dimensions, as measured by ImageJ, for area suggest that the calvarial structures moved in some way during the sequence of eight scans over six minutes. No data or observation from the present study is suggestive of mechanism of motion, be it due to bone compliance, due to viscoelastic properties of bone, or motion around cranial sutures. The finding of a mean area change of 122 mm^2 ^(Table 1) at that particular calvarial level could reflect the change in intracranial fluid volume identified in the NASA studies and postulated by Moskalenko et al [11] to account for most of the cranial bone motion implied in the PRM concept. In the NASA studies intracranial fluid volume was directly increased biomechanically in cadavers and in healthy human subjects by tilting them upside down with presumed gravitationally increased intracranial fluid volume, by pooling of either CSF or blood, or both.

It is recommended that future studies of this nature, using any imagery technology should use a stable marker in the image field to provide a reference point within the plane of reconstruction as well as orthogonal to it. The authors also acknowledge that MRI image resolution in the present study, while high quality and the best available at the research site, may not accurately identify changes less than the presumed 100 micron to 1.2 mm diameter changes found in other studies [4,5,9,19].

## Conclusion

The possible shortcomings notwithstanding, the data presented in the present study suggest that calvarial structures have motion characteristics that can be identified by MRI technology. It may be just a matter of time until increased resolution of MRI technology and image analysis provide the ability to examine more detailed areas of specific cranial bone motion and provide a reliable means for cranial bone motion research.

## Competing interests

The authors declare that they have no competing interests.

## Authors' contributions

HK and WC conceived of the study. HK obtained external funding and drafted the manuscript. WC obtained and coordinated support from Florida Hospital East Orlando for MRI studies. VG participated in study design, provided facility for preliminary imaging study and manuscript review for MRI technical accuracy. RP participated in the design and performed the imagery analysis and statistical analysis. All authors read and approved the final manuscript.
